# Molecular dynamic simulation for nanometric cutting of single-crystal face-centered cubic metals

**DOI:** 10.1186/1556-276X-9-622

**Published:** 2014-11-18

**Authors:** Yanhua Huang, Wenjun Zong

**Affiliations:** 1 Research Center of Laser Fusion, China Academy of Engineering Physics, Mianyang 621900, People’s Republic of China; 2 Center for Precision Engineering, Harbin Institute of Technology, P.O. Box 413, Harbin 150001, People’s Republic of China

**Keywords:** Nanometric cutting, Single crystal, Fcc metal, Molecular dynamics

## Abstract

In this work, molecular dynamics simulations are performed to investigate the influence of material properties on the nanometric cutting of single crystal copper and aluminum with a diamond cutting tool. The atomic interactions in the two metallic materials are modeled by two sets of embedded atom method (EAM) potential parameters. Simulation results show that although the plastic deformation of the two materials is achieved by dislocation activities, the deformation behavior and related physical phenomena, such as the machining forces, machined surface quality, and chip morphology, are significantly different for different materials. Furthermore, the influence of material properties on the nanometric cutting has a strong dependence on the operating temperature.

## Background

As one important enabling technology for achieving superior surface finish, single point diamond tool-based nanometric cutting has been widely applied in various industrial fields [1-3]. However, the lack of fundamental understanding of machining mechanisms greatly hinders the further development of the nanometric cutting machinability. In the conventional macroscopic machining where the edge radius of cutting tool (i.e., tool sharpness) is significantly smaller than the nominal depth of cut, the work piece is usually considered to be composed of continuous, isotropic, and defect-free materials. However, in the nanometric cutting, the work piece materials are mainly discrete, anisotropic, and not defect-free, because of the comparable edge radius of cutting tool with the nominal depth of cut. Consequently, the well-established conventional macroscopic machining mechanisms may be not be suitable for the interpretation of the nanometric cutting, and thus, a fundamental understanding of the nanometric cutting mechanisms is essentially required to facilitate the nanometric cutting technique.

The nanometric cutting mechanisms typically include elastic and plastic deformation of work piece material, friction and wear, machining forces, formation of machined surface and chip, etc. Since the nanometric cutting process is inherently discrete rather than continuous because of its ultra-small nominal depth of cut, which is of a few atomic layers, the experimental investigation of the nanometric cutting mechanisms is severely limited by the resolution of machining and measurement equipments, and the theoretical study based on the continuum mechanics such as finite element method cannot be used for the analysis of nanometric cutting process. Molecular dynamics (MD) has been demonstrated as one powerful tool for elucidating the nanometric cutting mechanisms, because it can trace the trajectories of individual atoms at a very short time step through the time integration of Newton's second law, which enables the monitoring of ongoing nanometric cutting process. Furthermore, workpiece with different microstructures and dimensions, as well as cutting tool with different geometries, can be constructed easily through modeling; and the machining parameters such as nominal depth of cut and cutting velocity can be adjusted conveniently for each nanometric cutting simulation. In the past few decades, many researchers have employed MD simulation to investigate the nanometric cutting mechanisms [4-10], and some typical work is outlined as follows. Komanduri et al. carried out MD simulations to analyze the effects of tool cutting edge radius and depth of cut in nanometric cutting [4]. Pei et al. further investigated the influence of rake angle on the nanometric cutting of copper [5]. Lai et al. studied the phase transformation during nanometric cutting of germanium [6]. Olufayo et al. addressed the brittle-ductile transition in nanometric cutting of mono-crystalline silicon [7]. Moreover, Promyoo et al. investigated the effects of tool rake angle and depth of cut on the cutting forces and chip formation in nanometric cutting [8].

The previous works have provided valuable insights into the nanometric cutting mechanisms. However, less attention has been paid to the influence of workpiece materials' properties on the nanometric cutting. It is well known that the nanometric cutting is a highly coupled process between workpiece material and cutting tool, because of the comparable edge radius of cutting tool with the nominal depth of cut. Consequently, the workpiece materials' properties may play a crucial role in the nanometric cutting process, in addition to the geometry of cutting tool. On the other side, it has been demonstrated that by alloying with aluminum, the properties and subsequent deformation behavior of copper can be significantly changed [11]. Thus, it is interesting to know whether the nanometric cutting mechanisms are also different for workpiece materials with different properties. In the current study, we perform MD simulations of nanometric cutting of single crystal aluminum and copper, which have the same face-centered cubic (FCC) lattice structure but different material properties.

## Methods

**Figure 1:**
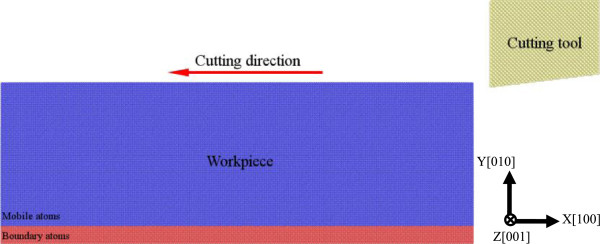
MD model of nanometric cutting.

In this work the LAMMPS code [12] is used to construct the MD model and perform MD simulations of nanometric cutting. Figure 1 shows that the MD model of nanometric cutting contains a workpiece and a straight diamond cutting tool. There are two types of atoms in the workpiece, as boundary atoms which are fixed in space and mobile atoms whose motion follows the Newtonian second law. To investigate the change of workpiece's temperature during nanometric cutting process, there is no thermostat atom in the workpiece. However, the influence of thermostat layer on the nanometric cutting process is further examined, in which the temperature of thermostat layer is controlled to be a constant value of 300 K by rescaling the velocities of thermostat atoms every ten steps. Periodic boundary condition is only applied in width direction. In this work, two kinds of workpiece materials are considered, i.e., single crystal copper and aluminum. Two workpieces have the same crystal orientation, as *X*:[100], *Y*:[010], and *Z*:[001] in length, height, and width directions, respectively. And the cutting motion is performed along the middle line of the sample, i.e., on the (010) surface and along the (-100) direction shown in Figure 1. Each workpiece has the same dimensions of 43.3, 14.5, and 29.0 nm in length, height, and width directions, respectively. Since the two materials have different lattice constants, i.e., 0.3615 nm for Cu and 0.405 nm for Al, the atom number for the modeled Cu workpiece (1542400) is larger than the Al workpiece (1130040). The straight diamond cutting tool treated as a rigid body has a cutting edge width of 7 nm, a rake angle of 0°, and a clearance angle of 7°. Two sets of parameters for Enterprise Asset Management (EAM) potential are used to describe Cu-Cu [13] and Al-Al [14] atomic interactions in the two types of workpiece. The EAM potential considers not only the pair-wise energy between atoms but also the embedding energy related to the ‘electron sea’ in which the atoms are embedded, which lead to accurate description of the properties of metallic materials. While there is no EAM parameters available between Cu and C atoms, Morse potential, which is relatively simple and computationally inexpensive compared to the EAM potential, is used for the atomic interactions between work piece and cutting tool [5,15].

In this work, the cutting velocity and the nominal depth of cut are fixed as 20 m/s and 2 nm, respectively. In order to investigate the chip formation mechanisms, the total cutting length is configured as 60 nm, which is larger than the length of work piece. Accordingly, the time step in any MD simulation is fixed as 5 fs to accelerate the simulation time, given the large cutting length. Two temperatures of 3 and 300 K are considered to evaluate the thermal fluctuation effect. While defect evolution plays an important role in the plastic deformation of metallic materials, identifying the types of defects is crucial for elucidating the nanometric cutting mechanisms. As compared to the centro-symmetry parameter [16] which provides values in different ranges for different types of defects, the technique of common neighbor analysis (CNA) provide unambiguous values for specific defects [17]. Therefore, the CNA is adopted to analyze the lattice defects generated within the work piece during nanometric cutting.

## Results and discussion

### Material-dependent nanometric cutting

**Figure 2:**
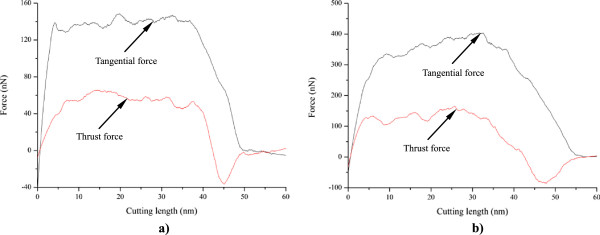
**Cutting force curves under an ambient temperature of 3 K. (a)** Aluminum and **(b)** copper.

**Figure 3:**
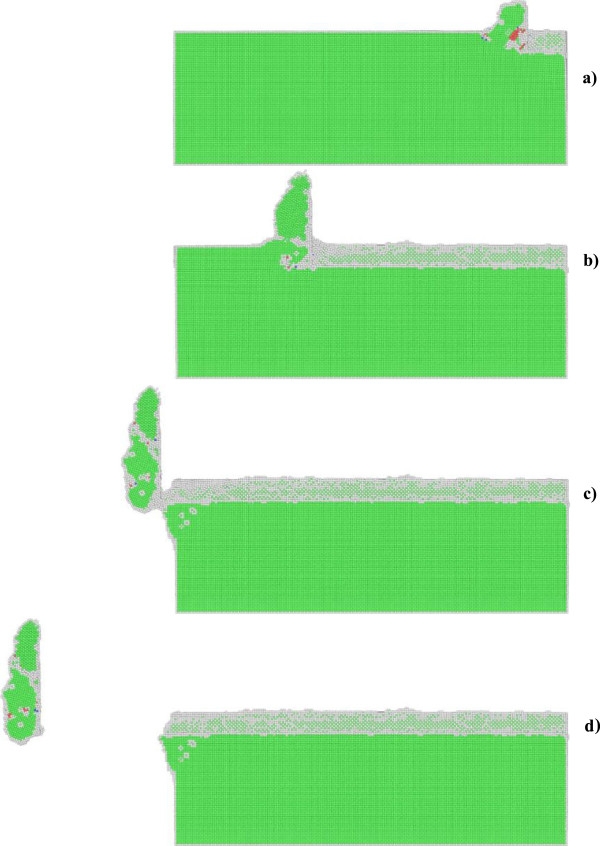
**Cross-sectional views of MD snapshots of nanometric cutting of single crystal aluminum at different cutting lengths. (a)** 6, **(b)** 30, **(c)** 47, and **(d)** 60 nm.

**Figure 4:**
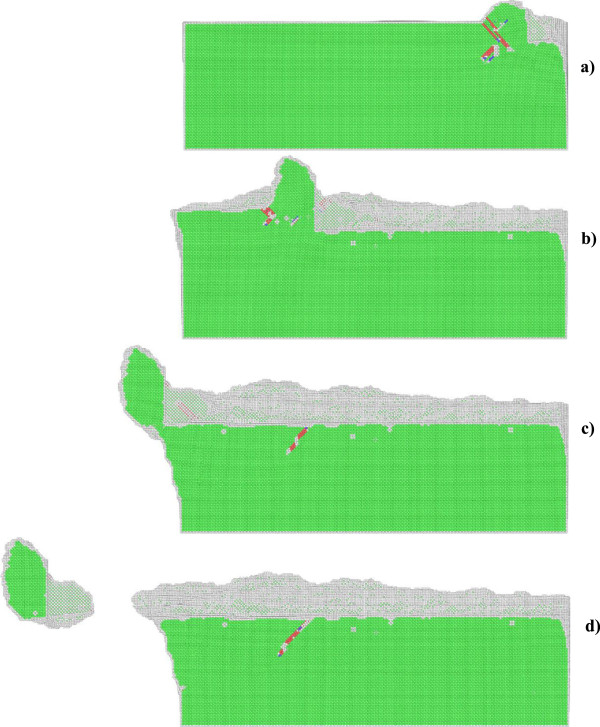
**Cross-sectional views of MD snapshots of nanometric cutting of single crystal copper at different cutting lengths. (a)** 6, **(b)** 30, **(c)** 47, and **(d)** 60 nm.

**Figure 5:**
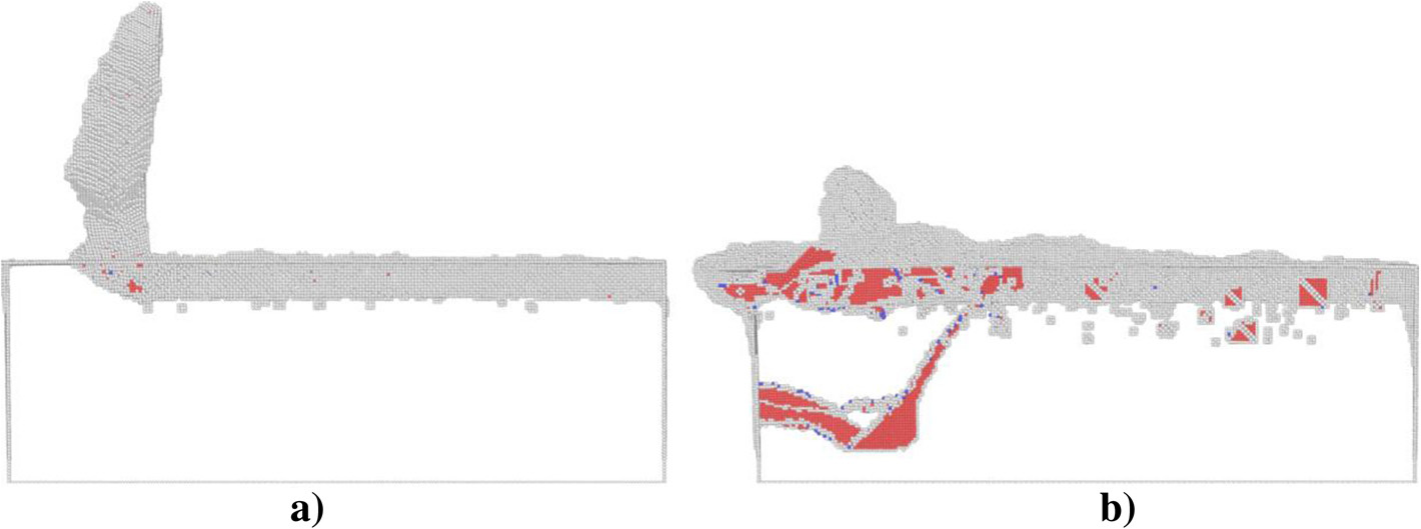
**Cross-sectional views of defect structures in work piece at a cutting length of 36 nm. (a)** Aluminum and **(b)** copper.

In order to elucidate underlying deformation mechanisms of metallic materials under nanometric cutting, MD simulations are first performed at low temperature of 3 K, at which the thermal vibration of atoms is negligible and identifying defects is unambiguous. Figure 2a,b shows variations of machining forces in nanometric cutting of single crystal aluminum and copper at the temperature of 3 K, respectively. The tangential force and thrust force are defined as the force component parallel to the [-100] direction and perpendicular to the (010) surface, respectively. For the cutting of aluminum, Figure 2a shows that in the initial cutting stage, the cutting tool is approaching towards the workpiece, both the tangential force and thrust force have negative values, which can be attributed to the adhesion effect between work piece and cutting tool. This adhesion effect is originated from the dominant attractive term of the pair-wise potential when the cutting tool has not reached the work piece. When the cutting tool starts to contact with the work piece, the material first deform elastically, as both the tangential force and thrust force increase linearly and rapidly. Figure 2a shows that when the cutting length reaches a critical value, being about 5 nm, the work piece material starts to surfer from the plastic deformation, and then the rapid increases of machining forces are terminated.In order to interpret the varying characteristics shown in the force-cutting length curves, Figures 3 and 4 present representative MD snapshots of single crystal aluminum and copper under nanometric cutting at different cutting lengths, respectively. Atoms are colored by their CNA values, as green, red and gray colors indicate fcc, hcp, and surface atoms, respectively. Figure 3a shows that at a cutting length of 6 nm, there are initial dislocations generated beneath the edge of cutting tool. Furthermore, there is considerable volume of material displaced by the movement of cutting tool. Resultantly, there is chip formed in the front of cutting tool. Figure 2a shows that after the aluminum work piece material starts to deform plastically, i.e., that the cutting length is larger than 5 nm, both the tangential force and thrust force respectively fluctuate around stable values of approximately 136 and 57 nN until the cutting length reaches up to a value of 37 nm, after which the cutting tool approaches the end of the work piece. Figure 3b shows that at a cutting length of 30 nm, there is considerable chip formed ahead of cutting tool, and the backside of the chip is straight due to the tight contact of formed chip with cutting tool rake face. Furthermore, the topography of the machined surface and subsurface damage seem to be very uniform, indicating the cutting process is quite stable. Upon further feeding, the cutting tool approaches the end of the work piece, and the accumulated chip starts to separate from the work piece. In this case, both the tangential force and thrust force decrease rapidly. Figure 3c shows that the separation of the chip from the work piece starts from its connection point with the machined surface. After separation, the chip keeps closely attached to the rake face of the cutting tool without further change, as pictorially shown in Figure 3d.Figure 2b shows that the variation characteristics of machining forces in the nanometric cutting of single crystal copper are generally consistent with the aluminum, as first rapid increase in the elastic deformation stage, followed by fluctuation around constant values when cutting process is stable, and finally decrease when the cutting tool approaches the end of the work piece. However, as compared to single crystal aluminum, Figure 2b clearly indicates that either the tangential force or the thrust force is considerably higher for single crystal copper. The constant values of tangential force and thrust force for single crystal copper are 374 and 130 nN, respectively.In addition to machining forces, the deformation behavior of the copper work piece appearing in the cutting process is also different from the aluminum work piece. It is known that the work piece material is displaced by the cutting tool in terms of formation of surface pile up around the cutting route and formation of chip in the front of the cutting tool rake face. The profile of surface pile up, including its distribution and accumulated volume, is strongly related with the machined surface quality such as surface roughness. Figure 4 shows that the machined surface quality of single crystal copper is significantly worse than single crystal aluminum, because of larger volume of surface pile up and non-uniform distribution of subsurface damage. Moreover, the chip morphologies in the two cutting processes are also different. While Figure 3 demonstrates that the aluminum workpiece has small chip volume and ‘brittle-like’ characteristic, the chip volume of the copper work piece is larger and has ‘ductile-like’ feature, as shown in Figure 4. Such difference indicates that the copper work piece suffers from much more severe plastic deformation in the cutting, which leads to the worse surface quality.Moreover, Figures 3 and 4 suggest that the plastic deformation mechanisms of the two work piece materials are also different. For example, there are more dislocations generated in the copper work piece than in the aluminum work piece. In order to characterize the difference in the deformation mechanisms, Figure 5a,b presents MD snapshots of defect structures in single crystal aluminum and copper at the same cutting length of 36 nm, respectively. Atoms are also colored by their CNA values. In order to represent the defect structures clearly in these two figures, the fcc atoms are eliminated. Figure 5a shows that for the aluminum dislocation activities are very little below either the machined surface or the cutting tool. In contrast, there are more dislocation activities observed in the copper work piece: first, there are many dislocation structures remained behind the cutting tool; second, there are considerable dislocations generated below the cutting tool, and the formation of dislocation loop structure due to dislocation reaction events can be observed.

**Figure 6:**
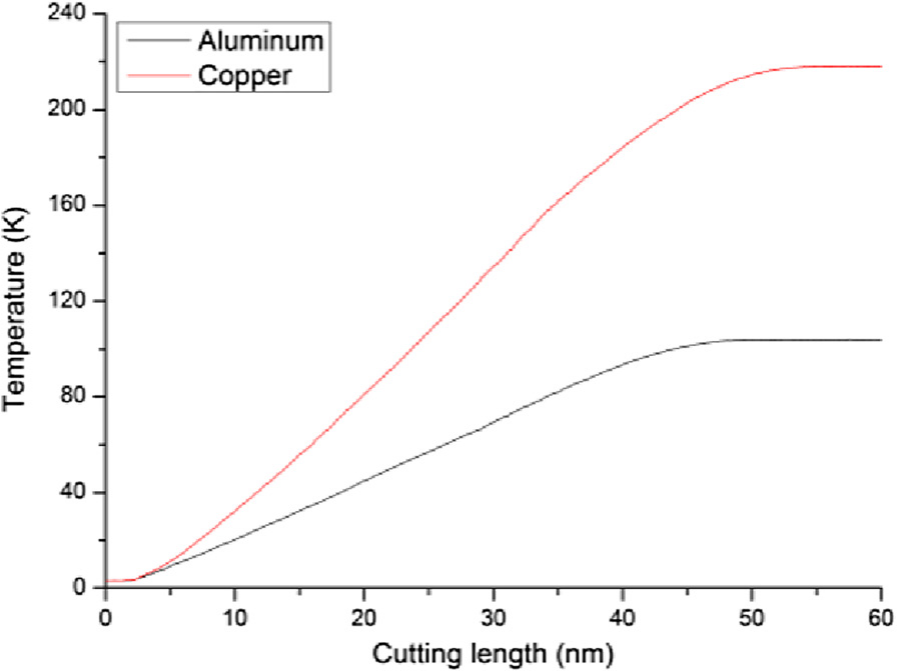
Variation of temperatures with cutting length in the aluminum and copper work pieces.

**Figure 7:**
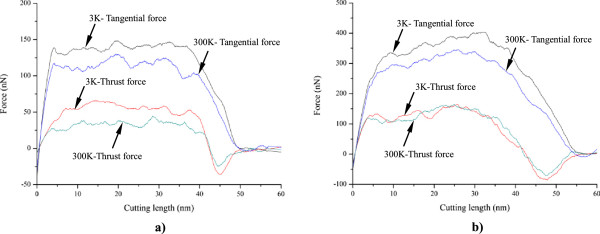
**Influence of ambient temperature on the cutting forces. (a)** Aluminum and **(b)** copper.

Besides the internal plastic deformation mechanisms, the chip morphologies of the two materials are also different. Figure 5a shows that for the aluminum work piece, the surface pile up is not significant but the chip volume is quite large, indicating that the displaced material is mainly transformed into chip. In reverse, for the copper work piece, the surface pile up is significant but chip volume is small, because of the considerable dislocation activities [18]. Figure 6 plots variations of temperature in the two work pieces with cutting length. It is found that the variation of temperature for each work piece is in accordance with the deformation behavior: the temperature first keeps constant value of 3 K when the material undergoes elastic deformation, followed by rapid increase in the plastic deformation regime because of the dissipation of heat introduced by the cutting action, and finally keeps constant value when cutting tool leaves the work piece. Although the initial temperatures in the two work pieces are the same as 3 K before cutting, the temperature in the copper work piece during cutting process is significantly higher than the aluminum work piece. To quantitatively characterize the temperature variation, the temperature increment is defined as the differential value of temperature between the constant values before and after cutting. It is found that the temperature increment of copper work piece is 215 K, which is significantly higher than the 100 K in aluminum work piece.Above analyses suggest that at a lower temperature of 3 K with negligible thermal fluctuation, nanometric cutting results in terms of deformation behavior, machining forces and achieved surface quality are heavily influenced by the properties of work piece material. In order to check whether the material properties-affected nanometric cutting has dependence on the operating temperature, MD simulations of nanometric cutting of single crystal aluminum and copper are also performed under a room temperature of 300 K. Figure 7a,b presents the force-cutting length curves in the nanometric cutting of single crystal aluminum and copper under the ambient temperatures of 3 K and 300 K, respectively. Figure 7a shows that for the aluminum, both the tangential force and thrust force under 300 K is smaller than that under 3 K. Moreover, the critical values of both tangential force and thrust force at which initial plastic deformation occurs in the work piece are also smaller for the higher temperature. For the copper, Figure 7b shows that the tangential force under 300 K is also lower than that under 3 K. Furthermore, the critical tangential and thrust forces associated with initial plastic deformation are both lower at high temperature. However, the constant value of the thrust force under 300 K is almost the same as that under 3 K. Since the initial plastic deformation is achieved by dislocation nucleation and motion for both the aluminum and copper, the critical forces for dislocation nucleation are lower at higher temperature, as thermal fluctuation under high temperature assists the cutting tool-induced metallic bonds breaking. Similarly, the tangential forces for both the aluminum and copper are also lower under higher temperature because of the higher thermal fluctuation.

**Figure 8:**
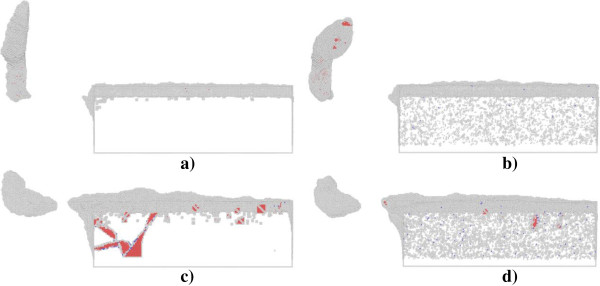
**Cross-sectional views of defect structures in work piece at a cutting length of 36 nm. (a)** Aluminum under 3 K, **(b)** aluminum under 300 K, **(c)** copper under 3 K, and **(d)** copper under 300 K.

Figure 8 presents defect structures in single crystal aluminum (top row) and copper (bottom row) after nanometric cutting under different ambient temperature. It is seen that there are considerable defects of atom clusters caused by the thermal effect in both the aluminum and copper work piece, which essentially lower the critical force associated with the initial plastic deformation. For the nanometric cutting of single crystal aluminum, Figure 8a,b demonstrates that the chip profile changes visibly at high temperature of 300 K, as the part exceeding the upper bound of the cutting tool is strongly curved, instead of keeping straight at low temperature of 3 K. However, the ambient temperature has negligible influence on the machined surface quality of the aluminum. For the nanometric cutting of single crystal copper, Figure 8c,d confirms that the dislocation activity is significantly decreased under the high temperature of 300 K, which leads to decreased volume of surface pile up. In this case, although the machined surface quality of the copper work piece seems to be improved to some extent, the chip morphology is almost invariable with the increment of the ambient temperature. Table 1 lists the temperature increments in the two kinds of work piece after the nanometric cutting operated under different temperatures. For each work piece, the temperature increment under low temperature of 3 K is larger than high temperature of 300 K. However, the temperature increment in the aluminum work piece is smaller than the copper work piece despite of the operating temperature. It should be noted that the temperatures presented in Table 1 are for the whole simulation system, instead of the cutting zone. Since the material removal occurs mainly in the vicinity of the cutting tool, the variation of the temperature in the cutting zone is significantly different from that in the entire simulation box.

**Table 1:** Temperature increments after nanometric cutting operated in different temperatures

**Material**	**Ambient temperature (K)**	**Temperature increment (K)**
Al	3	100
	300	76
Cu	3	215
	300	172

### Influence of thermostat layer

**Figure 9:**
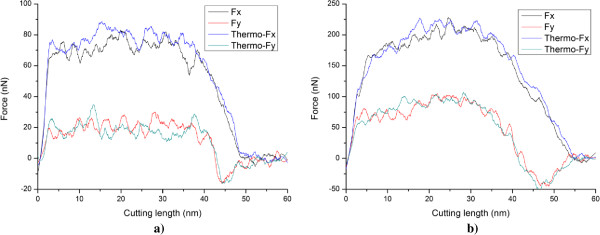
**Influence of thermostat layer on the cutting forces. (a)** Aluminum and **(b)** copper under a temperature of 300 K.

**Figure 10:**
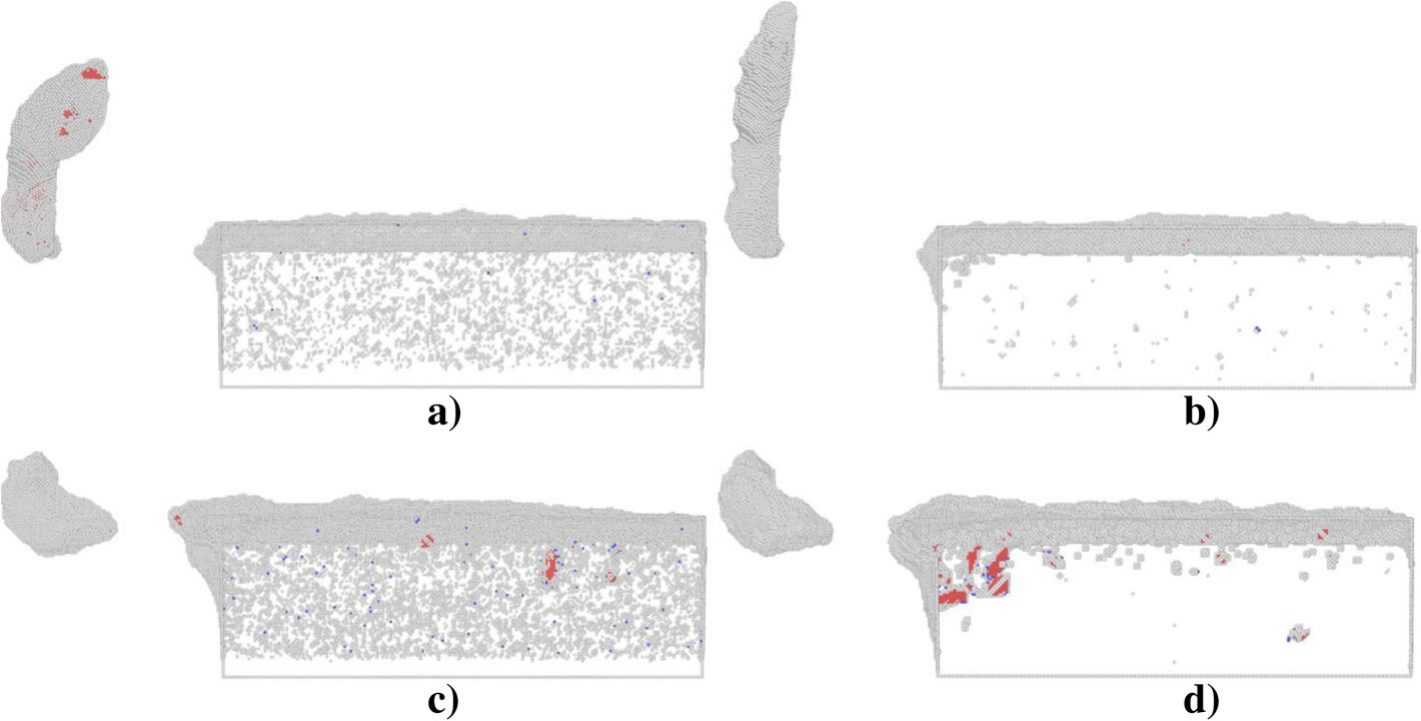
**Influence of thermostat layer on defect structures in work piece.** Aluminum **(a)** with and **(b)** without thermostat layer and copper **(c)** with and **(d)** without thermostat layer sampled at a cutting length of 36 nm and under an ambient temperature of 300 K.

The results in Table 1 are obtained by using the MD model without thermostat layer. In order to exam the influence of thermostat layer on the nanometric cutting, MD simulations using MD model with thermostat layer are further carried out. The temperature of thermostat layer is kept as constant value of 300 K. Figure 9 presents the force-cutting length curves in the nanometric cutting of single crystal aluminum and copper under the temperature of 300 K, using MD models with and without thermostat layer. The Fx and Fy denote the tangential force and thrust force, respectively. Figure 9 shows that for either the aluminum or copper work piece, the variations of forces are independent on the thermostat layer, in spite of the minor difference in the force fluctuations.Figure 10 presents defect structures in single crystal aluminum (top row) and copper (bottom row) after nanometric cutting using MD models with and without thermostat layer. Figure 10 indicates that with the thermostat layer, the number of thermal fluctuation-induced defects is dramatically decreased for both the aluminum and copper work piece. Particularly for the copper work piece with thermostat layer, there are more dislocations existed within the work piece. Figure 10 also shows that the chip profile is also affected by the thermostat layer. For the aluminum work piece, the chip profile is more straight and uniform for the MD model with thermostat layer than that without thermostat layer. However, the thermostat layer has trivial influence on the chip profile of the copper work piece.

## Conclusions

In this work, molecular dynamics simulations are conducted to investigate nanometric cutting of single crystal aluminum and copper. Based on the above analyses, some conclusions can be drawn as follows:

(1) The plastic deformation of fcc metals (aluminum and copper in the current study) under nanometric cutting is achieved by dislocation activities, i.e., dislocation nucleation and motion.

(2) The dislocation activities are less pronounced in the aluminum than that in the copper, which consequently results in smaller machining forces, fewer volume of surface pile up, smaller temperature increment, but larger volume of chip.

(3) The influence of material properties on the nanometric cutting has strong dependence on the operating temperature due to the inevitable thermal fluctuation.

(4) The presence of thermostat layer in the MD model has some influence on deformation behavior of workpiece, machining force, and chip profile.

## Competing interests

The authors declare that they have no competing interests.

## Authors’ contributions

YYH and WJZ designed the study, performed the molecular dynamics simulations, analyzed the data, and wrote the manuscript. Both authors read and approved the final manuscript.
